# Enzymatic hydrolysis of lupin protein isolates—Changes in the molecular weight distribution, technofunctional characteristics, and sensory attributes

**DOI:** 10.1002/fsn3.1139

**Published:** 2019-07-25

**Authors:** Katharina Schlegel, Katharina Sontheimer, Andrea Hickisch, Ali Abas Wani, Peter Eisner, Ute Schweiggert‐Weisz

**Affiliations:** ^1^ Chair of Aroma and Smell Research Department of Chemistry and Pharmacy Emil Fischer Center Friedrich‐Alexander‐Universität Erlangen‐Nürnberg Erlangen Germany; ^2^ Department Food Process Development Fraunhofer Institute for Process Engineering and Packaging IVV Freising Germany; ^3^ ZIEL – Institute for Food & Health, TUM School of Life Sciences Weihenstephan Technical University of Munich Freising Germany

**Keywords:** bitterness, enzymatic hydrolysis, functional properties, lupin allergens, SDS–PAGE

## Abstract

Enzymatic hydrolysis of lupin protein isolates (LPI; *Lupinus angustifolius* L.) was performed with nine different protease preparations to investigate their effect on technofunctionality, sensory properties, and the integrity of the proteins to estimate the reduction of the immunoreactivity. Alcalase 2.4 L, papain, and pepsin were most effective in the degradation of the α‐ and β‐conglutin examined by SDS–PAGE analysis, although the degree of hydrolysis only slightly increased. The technofunctional properties of LPI—solubility, emulsifying, and foaming activity—were improved by most of the proteolytic enzymes with the most impressive increase from 980% foam activity for LPI up to 3,614% foam activity for pepsin hydrolysate. The formation of bitterness, most likely linked to generation of bitter peptides, was pronounced in the Alcalase hydrolysate, while the other hydrolysates did not show an extensive increase in bitterness compared to the LPI. Other sensory attributes of the hydrolysates—with the exception of Alcalase treatment—were also very similar to the LPI. The results of this study show the potential of enzymatic degradation of LPI to modify the IgE‐reacting polypeptides and to improve the technofunctionality of the isolates and therefore their use as food ingredients.

## 1 INTRODUCTION

The demand for high‐quality plant proteins for applications in the food and feed sectors is increasing, and the search for alternative proteins has therefore expanded considerably in the last years. Lupins belonging to the Fabaceae family are widely grown in Europe and are a rich source of seed proteins (Arnoldi, Boschin, Zanoni, & Lammi, 2015). The protein content within lupin seeds can vary with 31% amounts in *Lupinus angustifolius* L. up to 44% in *Lupinus luteus* L. (Duranti, Restani, Poniatowska, & Cerletti, 1981). Lupin proteins exhibit valuable technofunctional properties and a well‐balanced sensory profile making them suitable ingredients for different kind of food products (Bader, Oviedo, Pickardt, & Eisner, 2011). The most abundant storage proteins in lupin seeds are the globulins, which comprise two major protein types, β‐conglutin (7*S* globulin, vicilin‐like protein) and α‐conglutin (11*S* globulin, legumin‐like protein) and minor components, γ‐conglutin and δ‐conglutin (Duranti et al., 1981). β‐Conglutin is known as a major allergen (molecular weight of ~55–61 kDa) and classified as a recognized allergen with the code Lup an 1.0101 by the International Union of Immunological Societies allergen nomenclature subcommittee for *L. angustifolius* L. (Ballabio et al., 2013; Goggin, Mir, Smith, Stuckey, & Smith, 2008). Although the prevalence of sensitization and allergenic reaction is less known in the general population and as lupin becomes more popular as an alternative protein source for human consumption, the increased demand for the proteins may expose more consumers to lupin antigens (Jimenez‐Lopez et al., 2018). Lupin and its products have been included in Annex IIIa of Directive 2000/13/EC, which lists ingredients that must be declared on food labeling. The known cases of lupin allergies have mainly been reported in patients with allergies to other legumes such as soybean, pea, lentil, chickpea, and particularly peanut (Jappe & Vieths, 2010), probably due to cross‐reactions to structurally similar proteins including similar epitope regions from other legume species (Jimenez‐Lopez et al., 2018).

The increasing prevalence of food allergies and protein sensitization has been addressed with several attempts to reduce the allergenic potential of food proteins to mitigate allergenic reactions in susceptible individuals (Chizoba Ekezie, Cheng, & Sun, 2018). Allergens can be inactivated by heat treatment, but this also affects other proteins and has a dramatic effect on food quality. Nonthermal technologies including pulsed light, high‐pressure processing, gamma irradiation, cold plasma technology, ultrasonication, and pulsed electric fields were also described (Chizoba Ekezie et al., 2018), but most of these methods do not achieve the complete inactivation of allergens or have not been studied sufficiently. Another promising approach for the removal of allergens is their enzymatic‐assisted hydrolysis.

Extensive or mild protein hydrolysis can be used to prepare hypoallergenic foods, particularly those based on soybean proteins (Lqari, Pedroche, Girón‐Calle, Vioque, & Millán, 2005). Furthermore, there is a potential impact on their functional properties, such as protein solubility, foaming, and emulsifying capacity (EC; Chabanon, Chevalot, Framboisier, Chenu, & Marc, 2007; Hall, Jones, O'Haire, & Liceaga, 2017; Lqari et al., 2005; Meinlschmidt, Schweiggert‐Weisz, Brode, & Eisner, 2016; Meinlschmidt, Sussmann, Schweiggert‐Weisz, & Eisner, 2016; Purschke, Meinlschmidt, Horn, Rieder, & Jäger, 2018). Moreover, protein hydrolysis can also affect the sensory properties of the protein ingredient. In particular, the formation of a bitter taste restricts the use of food ingredients (Spellman, O'Cuinn, & FitzGerald, 2004). The bitterness of protein hydrolysates primarily reflects the release of low molecular weight peptides containing hydrophobic amino acid residues (Cho, Unklesbay, Hsieh, & Clarke, 2004; Fu, Liu, Hansen, Bredie, & Lametsch, 2018; Kim & Li‐Chan, 2006; Matoba & Hata, 1972) and correlates positively with the degree of hydrolysis (DH) when DH values are very low (Fu et al., 2018; Newman et al., 2014).

In the case of soy proteins, several studies showing the impact of protease treatment on technofunctional and sensory properties as well as their allergenic potential could be found in the literature. However, the influences of enzymatic hydrolysis on lupin proteins have only scarcely been investigated up to now. Few studies on lupin proteins targeted either change in their technofunctional properties after enzymatic treatment or the reduction of their allergenic potential (Czubinski, Montowska, Pospiech, & Lampart‐Szczapa, 2017; Lqari et al., 2005; Raymundo, Empis, & Sousa, 1998). Sormus de Castro Pinto, Neves, and Machado de Medeiros (2009) estimated the decrease in antigenic activity of the globulins of lupin due to enzymatic hydrolysis with pepsin and trypsin, while Álvarez‐Álvarez et al. (2005) studied the allergen characterization of lupin seeds after different boiling treatments. To the best of our knowledge, a study simultaneously investigating the impact of proteolysis on technofunctional and sensory properties of lupin proteins as well as a first estimation of their allergenic potential is not available in literature. Therefore, the objective of this study was to determine the effectiveness of different proteases for the depletion or elimination of major IgE‐reacting polypeptides in *L. angustifolius* cultivar Boregine. We evaluated the technofunctional properties of lupin protein hydrolysates and the impact of hydrolysis on the sensory qualities of a lupin protein isolate (LPI).

## 2 MATERIALS AND METHODS

### 2.1 Raw materials and chemicals

Lupin (*L. angustifolius* cultivar Boregine) seeds were purchased from Saatzucht Steinach GmbH & Co KG. The sources and properties of the enzymes are listed in Table 1.

**Table 1:** Sources and properties of the enzymes used in this study

Enzyme	Type	Biological source	Supplier
Alcalase^®^ 2.4 L FG	Serine endopeptidase	*Bacillus licheniformis*	Novozymes A/S (Bagsvaerd, Denmark)
Neutrase^®^ 0.8 L	Metallo endopeptidase	*Bacillus amyloliquefaciens*	Novozymes A/S
Flavourzyme^®^ 1000 L	Amino endopeptidase and exoprotease	*Aspergillus oryzae*	Novozymes A/S
Protamex^®^	Serine endopeptidase	*Bacillus licheniformis*, *Bacillus amyloliquefaciens*	Novozymes A/S
Papain	Cysteine endopeptidase	Papaya (*Carica* sp.) latex	AppliChem GmbH (Darmstadt, Germany)
Pepsin	Aspartic endopeptidase	Porcine (*Sus domesticus*) gastric mucosa	Merck KGaA (Darmstadt, Germany)
Corolase^®^ 7089	Metallo and serine endopeptidase	*Bacillus subtilis*	AB Enzymes GmbH (Darmstadt, Germany)
Corolase^®^ N	Metallo and serine endopeptidase	*Bacillus subtilis*	AB Enzymes
Protease N‐01	Serine endopeptidase	*Bacillus subtilis*	ASA Spezialenzyme GmbH (Wolfenbüttel, Germany)

### 2.2 Preparation of lupin protein isolate

Lupin protein isolate was prepared from lupin seeds. Briefly, the seeds were dehulled using an underrunner disk sheller (Streckel & Schrader KG) and then separated and classified using an air‐lift system (Alpine Hosakawa AG). The dehulled seeds were passed through a counter‐rotating roller mill (Streckel & Schrader KG). The resulting flakes were deoiled in *n*‐hexane in a 1.5‐m^3^ percolator (e&e Verfahrenstechnik GmbH). The solvent was removed via flash desolventation (hexane with 400–500 mbar) and steam desolventation finally. The processed flakes were then suspended in 0.5 M HCl (pH 4.5) at a 1:8 (w/w) ratio and then stirred for 1 hr at room temperature. The flakes were recovered in a decanter centrifuge (5,600 *g*, 4°C, 60 min) (GEA Westfalia Separator Deutschland GmbH) and the supernatant containing the γ‐conglutin fraction was discarded. The acid pre‐extracted flakes were dispersed in 0.5 M NaOH (pH 8.0) at a 1:8 w/w ratio. After extraction for 60 min, the suspension was centrifuged (5,600 *g*, 20°C, 60 min) and the supernatant contained the main storage protein fractions, α‐conglutin and β‐conglutin. Aliquots of 0.5 M HCl were added to the supernatant at room temperature to facilitate the protein precipitation at a pH of 4.5. The precipitated proteins were separated by centrifugation at 5,600 *g* for 130 min (GEA Westfalia Separator Deutschland GmbH) and then neutralized (0.5 M NaOH), pasteurized (70°C, 10 min), and spray dried (APV Anhydro AS Drying & Evaporation).

### 2.3 Enzymatic hydrolysis of LPI

Enzymatic hydrolysis of LPI was carried out in a 4 L thermostatically controlled reaction vessel, as previously described by Meinlschmidt, Sussmann, et al. (2016). Proteases were chosen according to Meinlschmidt, Sussmann, et al. (2016), where promising results were achieved in the degradation of β‐conglycinin and glycinin in soy. The reaction conditions were selected based on the suppliers' application sheets and shown in Table 2. The reaction conditions for papain were selected according to Tsumura, Saito, Kugimiya, and Inouye (2004), who observed a gradually increased degradation of β‐conglycinin in soy protein isolate by increasing reaction temperature above 60°C and substantially resistant glycinin at hydrolysis temperatures below 80°C. For LPI hydrolysis, the protein isolate was dispersed with an Ultraturrax (IKA‐Werke GmbH & Co. KG) for 1 min at 5,000 rpm in deionized water to achieve a protein concentration of 50 g/kg. Enzyme‐specific temperatures and pH values were adjusted prior to the addition of the protease preparations. The amount of enzyme preparation added is shown in Table 2. After incubation, the suspension was continuously stirred at a controlled pH and temperature for 2 hr. To avoid further hydrolysis, the reaction was terminated by heating to 90°C for 20 min, cooled down to room temperature, and neutralized (pH 7.0). Control LPI dispersions (no enzyme) were prepared under the same conditions and inactivation treatment. Samples were frozen at −50°C and lyophilized (BETA 1–8, Martin Christ Gefriertrocknungsanlagen GmbH). All experiments were performed in duplicate.

**Table 2:** Protease preparations for LPI hydrolysis

Protease	E/S (%)	Temperature (°C)	pH value
Alcalase 2.4 L	0.5	50	8.0
Papain	0.2	80	7.0
Neutrase 0.8 L	0.5	50	6.5
Protease N‐01	0.5	55	7.2
Flavourzyme 1000 L	0.5	50	6.0
Protamex	0.5	60	8.0
Corolase 7089	0.5	55	7.0
Pepsin	0.5	50	2.0
Corolase N	0.5	50	7.0

Abbreviation: E/S, enzyme‐to‐solution ratio.

### 2.4 Chemical composition

The protein content was calculated based on the nitrogen content, which was determined using a Nitrogen Analyzer FP 528 (Leco Corporation) according to the Dumas combustion method (AOAC 968.06). A factor of N × 5.8 was used to calculate the protein content according to Mosse, Huet, and Baudet (1987). The dry matter was analyzed according to AOAC methods 925.10 in a TGA 601 thermogravimetric system (Leco Corporation) at 105°C.

### 2.5 Protein analysis

#### 2.5.1 Degree of hydrolysis

The DH was determined for each hydrolysate in duplicate using the o‐phthaldialdehyde (OPA) method as previously described (Nielsen, Petersen, & Dambmann, 2001) with serine as the standard (Adler‐Nissen, 1986). The percentage of DH was calculated using formula:

$$DH=h/h_{tot}\times 100$$

where *h*_tot_ is the total number of peptide bonds per protein equivalent with a factor of 7.8 (based on soybean protein) according to Adler‐Nissen (1986), and *h* is the number of hydrolyzed bonds.

#### 2.5.2 SDS–PAGE

The molecular weight distribution of the lupin protein hydrolysates was determined by sodium dodecyl sulfate–polyacrylamide gel electrophoresis (SDS–PAGE) as described by Laemmli (1970). Lupin protein isolate, hydrolysate, and control samples were resuspended in 1 ml loading buffer (0.125 mol/L Tris–HCl, 4% SDS (w/v), 20% glycerol (v/v), 0.2 mol/L DDT, 0.02% bromophenol blue, pH 6.8), dissolved for 15 min at 30°C in an ultrasonic bath, and boiled for 5 min at 95°C to cleave noncovalent bonds. Following centrifugation at 13,000 *g* for 10 min (Mini Spin, Eppendorf AG), an aliquot of the supernatant was transferred to a fresh tube and supplemented in a ratio of 1:10 with loading buffer (see above). We then transferred 10 µl aliquots (5 mg/ml protein) into the wells of precast 4%–20% polyacrylamide gels (Bio‐Rad Laboratories). The samples were separated for 40 min at 200 V (60 mA, 100 W) (Amersham Biosciences Europe GmbH) at room temperature in a vertical electrophoresis cell (Bio‐Rad Laboratories). Precision Plus Protein Unstained Standard with molecular weight of 10–250 kDa (Bio‐Rad Laboratories) run alongside as size markers, and the protein subunits were visualized using a Gel Doc™ EZ Imager system (Bio‐Rad Laboratories). The molecular weight distribution was determined using Image Lab software (Bio‐Rad Laboratories).

#### 2.5.3 Fractionation of LPI using anion exchange chromatography

For the fractionation of LPI, anion exchange chromatography was applied according to Melo, Ferreira, and Teixeira (1994) and Sirtori, O'Kane, Brambilla, and Arnoldi (2008) using a DEAE Sepharose™ Fast Flow Column (1.6 cm, 15 ml, GE Healthcare). The column was equilibrated with 0.1 M Tris–HCl (pH 8.2) and was loaded with 2 ml 5% (w/v) LPI solution. For elution of the fractions, the following gradient was used: 0.05, 0.10, 0.15, 0.20, 0.25 M NaCl in 0.1 M Tris–HCl (pH 8.2). β‐Conglutin was eluted at a salt concentration of 0.15 M NaCl, followed by α‐conglutin eluted at 0.20–0.25 M NaCl. The fractions were collected and desalted by dialysis, and its purity was confirmed by SDS–PAGE as described before.

### 2.6 Technofunctional properties

#### 2.6.1 Protein solubility

The solubility (%) of the LPI and its hydrolysates was determined in duplicate over the pH range 4.0–9.0 (Morr et al., 1985). For each measurement, 1.5 g of protein was suspended in 50 ml 0.1 M NaCl. The pH was adjusted with 0.1 M NaOH or 0.1 M HCl, and the suspension was stirred for 1 hr at room temperature. Nondissolved fractions of the samples were separated by centrifugation (20,000 *g*, 15 min, room temperature), and the supernatants were passed through Whatman No. 1 filter paper to remove any remaining particulates. The protein content of the supernatant was determined by nitrogen analysis according to AOAC 968.06 as above, and the protein solubility was calculated as follows:

$$\text{Protein}\;\text{solubility}\;\left[\%\right]=\frac{\text{initial}\;\text{volume}\;\left[\text{ml}\right]\times \text{protein}\;\text{content}\;\text{in}\;\text{supernatant}\;\left[\frac{\text{mg}}{\text{ml}}\right]}{\text{sample}\;\text{mass}\;[\text{mg}]\times \text{protein}\;\text{content}\;[\%\text{dry}\;\text{matter}]\times \text{dry}\;\text{matter}\;[\%]}\times 100\;$$

#### 2.6.2 Foaming properties

Foaming activity was determined in duplicate as recommended by Phillips, Haque, and Kinsella (1987). 100 ml of a 5% (w/w) protein solution at pH 7.0 and room temperature was whipped for 8 min in a Hobart 50‐N device (Hobart GmbH). The increase in volume after whipping was used to calculate the foam activity. The foam density (g/L) was quantified by weighing a selected amount of foam volume and was reported as a ratio of foam volume to foam weight. The loss of foam volume after 1 hr was defined as the foaming stability (%).

#### 2.6.3 Emulsifying capacity

Emulsifying capacity was determined at pH 7.0 according to the method described by Wang and Johnson (2001). Duplicate samples were dispersed in deionized water (1% w/w), adjusted to pH 7.0, and stirred with an Ultraturrax at 18°C. Rapeseed oil was added using a Titrino 702 SM titration system (Metrohm GmbH & Co. KG) at a constant rate of 10 ml/min until a phase inversion was detected by continuous measurement of the electrical conductivity using an LF 521 meter fitted with a KLE1/T electrode (Wissenschaftlich‐technische Werkstätten GmbH). The volume of oil needed to achieve the phase inversion was used to calculate the EC (ml oil per g sample).

### 2.7 Sensory analysis of protein hydrolysates

#### 2.7.1 Training of the panel

A sensory panel of 10 persons was trained to evaluate bitterness using the DIN 10,959 threshold tests with caffeine solutions of 0, 0.025, 0.05, 0.075, 0.1, 0.0125, 0.15, 0.175, 0.2, and 0.225 g/L. An Alcalase 2.4 L hydrolysate was also provided for the training session as described by Meinlschmidt, Sussmann, et al. (2016). For the Alcalase 2.4 L hydrolysate, a 5% LPI dispersion was hydrolyzed with Alcalase 2.4 L (0.5% (w/w) at pH 8.0, 60°C) for 3 hr. The hydrolysate was then neutralized (3 M NaOH) and freeze‐dried, and the dried hydrolysate was dissolved in tap water to prepare solutions of 0.05%, 0.1%, 0.25%, 0.5%, 1.0%, 1.5%, 2.5%, 3.5%, 4.5%, 5.5%, 6.5%, 7.5%, and 8.5% (w/w).

#### 2.7.2 Descriptive analysis

Sensory analysis was performed in a laboratory compliant with international standards. A sample of LPI solution (1% w/w) and a sample of LPI Alcalase 2.4 L hydrolysate solution (1% w/w) were presented to the sensory panel in glass vessels (capacity 140 ml) for retronasal evaluation. The panelists were not informed about the sample description during the analysis of taste and retronasal attributes. Detected taste and flavor attributes of each panelist were collected, and the final attributes were selected based on the frequency of detection. For the taste attributes, the following references were used to determine the selected sensory attributes: bitter (1% LPI Alcalase 2.4 L hydrolysate), salty (0.5% NaCl), and a trimeric astringent perception test. The flavor references included the attributes metallic (tr‐4,5‐epoxy‐(*E*)‐2‐decenal), oatmeal‐like (oatmeal), earthy, moldy, beetroot‐like (geosmin), fatty, cardboard‐like ((*E*)‐2‐nonenal), grassy (hexanal), pea‐like (3‐s‐butyl‐2‐methoxypyrazine), and cooked potato‐like (3‐(methylthio‐)propanal).

#### 2.7.3 Sample preparation

For sample evaluation, 1% (w/w) solutions of the LPI and LPI hydrolysates, respectively, and tap water were prepared by stirring. Each panelist received five samples (20 ml) per session in plastic cups with random three‐digit codes. The bitter and salty references were prepared as thoroughly stirred solutions of 1% (w/w) LPI Alcalase 2.4 L hydrolysate and a 0.5% (w/w) NaCl.

#### 2.7.4 Sensory evaluation

Each sample was evaluated by the trained panel, with tap water and flavorless crackers used to neutralize the sensory attributes between each sample. The intensity of each attribute was scored on an unstructured 10‐cm line between *not noticeable* at the left and *very strong* at the right. The sensory evaluation of the bitter and salty tastes and the trimeric astringent perception test were performed with a nasal clamp to suppress all retronasal sensations. For the bitter and salty reference, the solutions of 1% (w/w) LPI Alcalase 2.4 L hydrolysate and a 0.5% (w/w) NaCl were used. We used scent sticks with the appropriate flavor solution for orthonasal perception prior to each retronasal flavor perception test.

### 2.8 Statistical analysis

Data were analyzed using OriginPro 2016 for Windows (Origin Lab Corporation). Results are expressed as mean ± *SD*. One‐way analysis of variances (ANOVA) was applied, and Tukey's honestly significant difference post hoc test was used to determine the significance of differences between samples, with a threshold of *p* < .05.

## 3 RESULTS AND DISCUSSION

The LPI and its proteolytic hydrolysates (containing a dry matter of 90% and a protein content of 92% using the conversion factor of 5.8) were analyzed for changes in DH and molecular weight distribution (SDS–PAGE) to get a first indication of the reduction in the allergenic potential. Furthermore, protein solubility, EC, and foaming as well as sensory attributes were determined as those are important for the proteins as food ingredients. The results are discussed in detail below.

### 3.1 Effects on protein degradation

#### 3.1.1 Degree of hydrolysis

The enzymatic hydrolysis of proteins cleaves the peptide bonds to form peptides. As many proteins can cause an allergic reaction, the proteolytic hydrolysis of protein epitopes might be a promising technique to reduce the allergenic potential of a protein as presented in several studies (Meinlschmidt, Schweiggert‐Weisz, et al., 2016; Sormus de Castro Pinto et al., 2009). The DH was determined to get an indication of the integrity of the protein after 2 hr of hydrolysis. The results are shown in Table 3. The average DH for nonhydrolyzed LPI was 0.88%, which increased to the highest DH of 9.05% after the treatment with Alcalase 2.4 L. Lower DH values of 6.90%, 6.48%, and 5.07% were observed following the treatments with Flavourzyme 1000 L, Protamex, and Corolase 7089, respectively. Protease N‐01 was the least efficient proteolytic enzyme, achieving a DH of 2.38% after 2 hr of hydrolysis. Similar observations were already reported for the hydrolysis of soy protein isolate (Meinlschmidt, Sussmann, et al., 2016). These results are marginally higher than the values we found in this study. In control reactions without enzymes, no increase in the DH was observed.

**Table 3:** Degree of hydrolysis (DH) (%) of hydrolyzed LPI obtained after different protease treatments

Protease used for hydrolysis	Degree of hydrolysis (%)	
	Time of hydrolysis	
	2 hr	2 hr (control reactions without enzymes)
Alcalase 2.4 L	9.05 ± 0.46^a^	1.32 ± 0.26^a^
Papain	2.61 ± 0.66^b^	0.82 ± 0.10^a,b^
Neutrase 0.8 L	4.67 ± 0.13^c,f^	0.85 ± 0.08^a,b^
Protease N‐01	2.38 ± 0.33^b^	0.82 ± 0.06^a,b^
Flavourzyme 1000 L	6.90 ± 0.17^d^	0.94 ± 0.15^a,b^
Protamex	6.48 ± 0.10^d^	0.72 ± 0.01^b^
Corolase 7089	5.07 ± 0.11^c^	0.74 ± 0.03^b^
Pepsin	3.37 ± 0.26^e^	0.66 ± 0.01^b^
Corolase N	4.31 ± 0.10^f^	0.85 ± 0.06^a,b^

Note

The data are expressed as mean ± *SD* (*n* = 4). Values followed by different letters in a column indicate significant differences between groups (*p* < .05).

### 3.2 Molecular weight distribution (SDS–PAGE)

**Figure 1:**
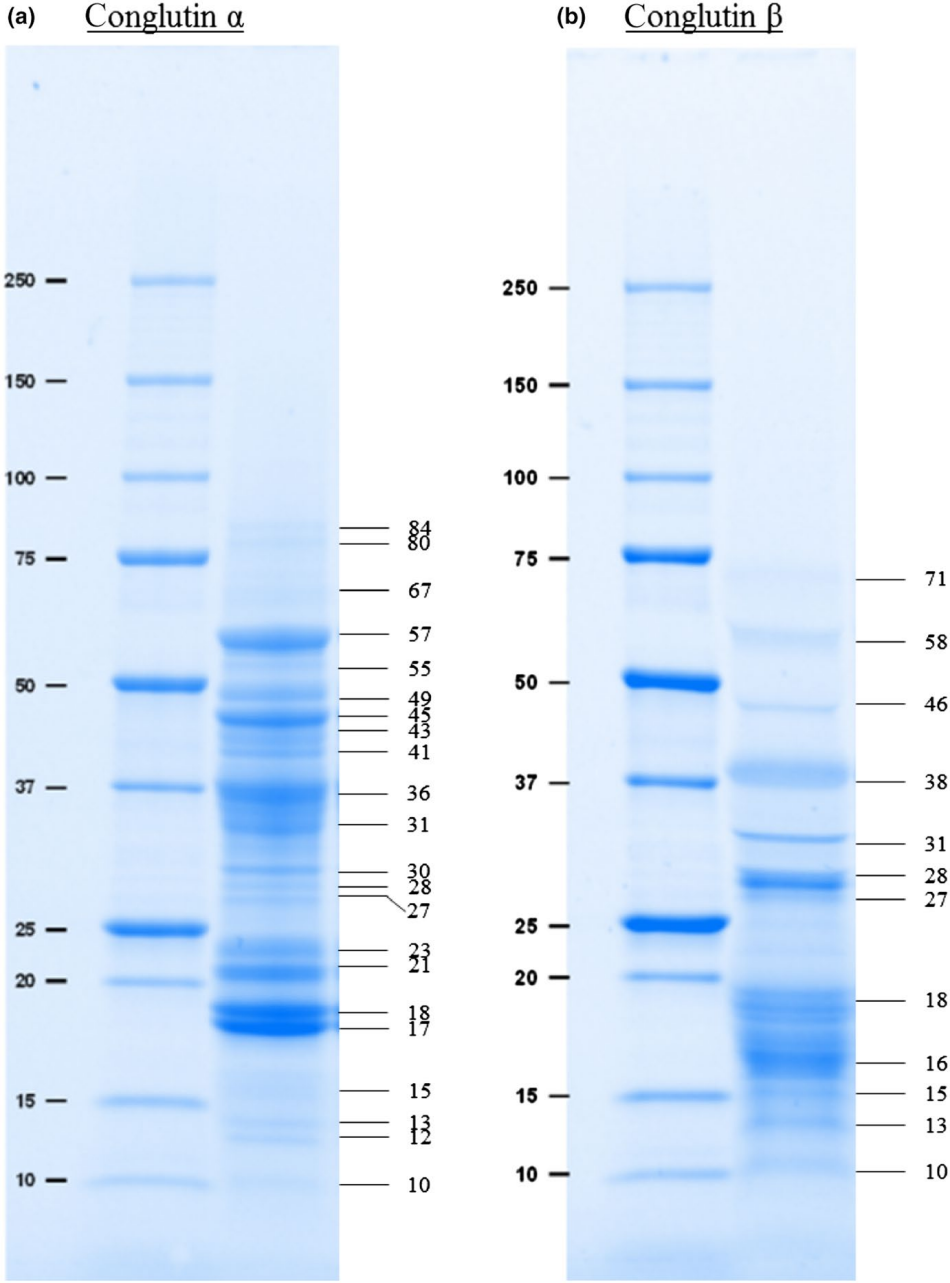
Molecular weight (kDa) of native α‐conglutin (a) and β‐conglutin (b) in *Lupinus angustifolius* L. cultivar Boregine as determined by SDS–PAGE under reducing conditions

Besides the DH, the molecular weight distribution of the LPI and its hydrolysates by means of SDS–PAGE was also used for the estimation of the protein integrity. To corroborate the presence of α‐conglutin and β‐conglutin in the LPI and to facilitate the interpretation of the hydrolysis results, the lupin protein was therefore separated into the two protein fractions using anion exchange chromatography. The SDS–PAGE profile of the individual fractions is shown in Figure 1. Under reducing conditions, native α‐conglutin of *L. angustifolius* cultivar Boregine was composed of low molecular weight (10–23 kDa), medium molecular weight (27–36 kDa), and high‐molecular‐weight (41–84 kDa) polypeptides (Figure 1a). β‐Conglutin also consisted of polypeptides with molecular weights of 10, 13, 15, 16, and 18 kDa as well as additional, heavier polypeptides with molecular weights of 27, 28, 31, 38, 46, 58, and 71 kDa (Figure 1b). These observations are similar to Ballabio et al. (2013), Blagrove and Gillespie (1975), Goggin et al. (2008) and Monteiro, Freitas, Rajasekhar, Teixeira, and Ferreira (2010). However, there were slight deviations in the distribution of bands and molecular weight. This can be attributed to different species, origin, and seasonal fluctuations.

**Figure 2:**
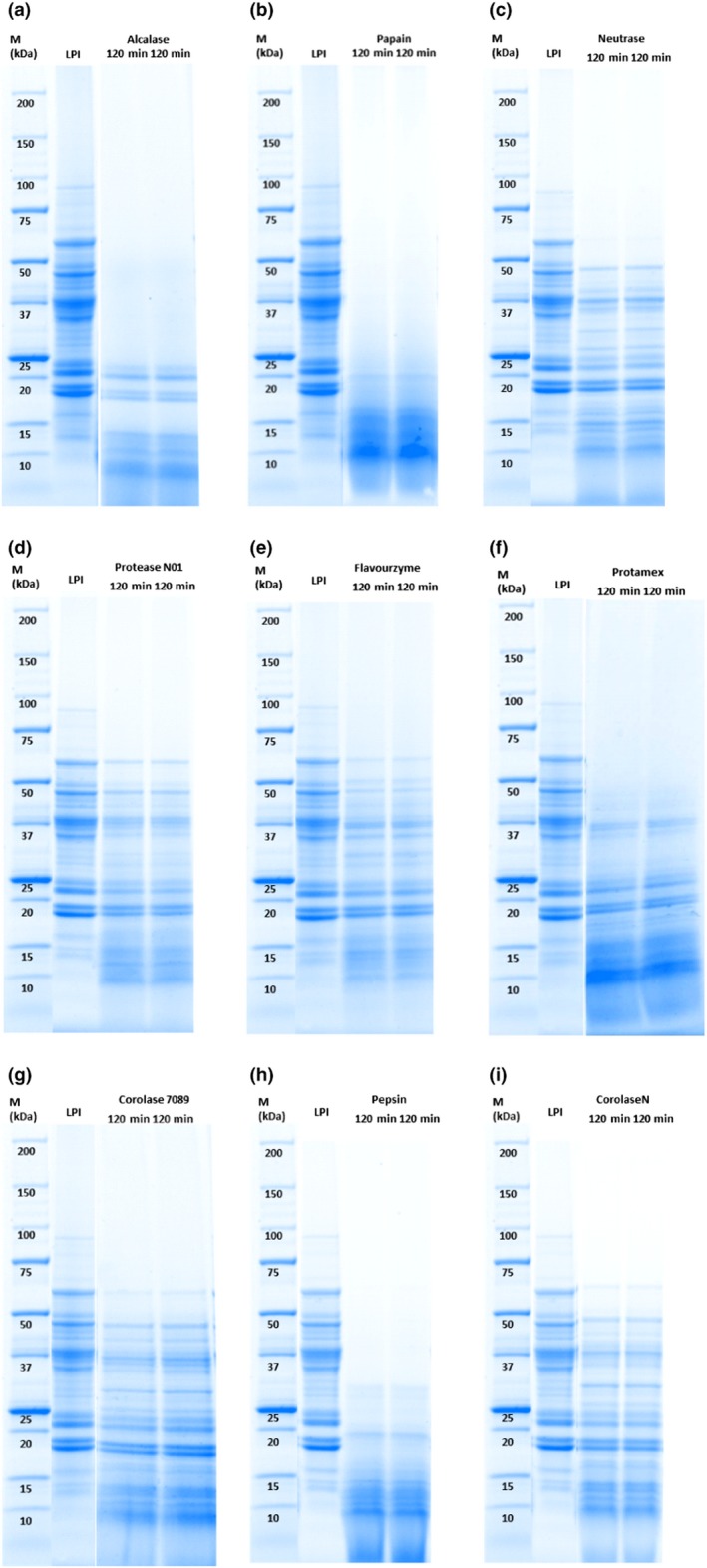
Peptide band profiles in LPI hydrolysates produced by treatment with different proteases as determined by SDS–PAGE under reducing conditions in duplicate

Treatment with Alcalase 2.4 L, papain, pepsin, and Protamex resulted in prominent changes in the SDS–PAGE profile, reflecting the extensive hydrolysis of both allergens (Figure 2(a,b,f,h)). With the exception of light subunits of pepsin (27–30 kDa) and Protamex (31–38 kDa) hydrolysates, the polypeptides were hydrolyzed to smaller fragments, with molecular weights below 23 kDa. Alcalase 2.4 L preparation from *Bacillus licheniformis* and Protamex preparation from *Bacillus licheniformis and Bacillus amyloliquefaciens* are classified as serine endopeptidases*,* wherein serine acts as the nucleophilic amino acid at the active site of the enzyme, which cleave peptide bonds in proteins. The SDS–PAGE results suggest that both serine endopeptidase preparations are able to hydrolyze the high‐molecular‐weight fractions of α‐conglutin and β‐conglutin. In addition, Alcalase 2.4 L endopeptidase was also effective in the degradation of medium molecular weight fractions of LPI. Papain is classified as cysteine endopeptidase with specific substrate preferences for bulky hydrophobic or aromatic residues. The Papain preparation is composed of endo‐ and exoprotease activities being highly efficient in the hydrolysis of hydrophobic or aromatic residues in high, medium, as well as low molecular weight polypeptides of LPI. Pepsin is an aspartic endopeptidase that specifically cleaves bonds in peptides which have at least six residues in length with hydrophobic residues. According to the SDS–PAGE results, pepsin‐specific compounds appear to be present in the α‐conglutin and β‐conglutin polypeptides above 23 kDa in LPI. Similar SDS–PAGE results could be obtained by Meinlschmidt, Sussmann, et al. (2016) with soy protein, Purschke et al. (2018) with insect protein and Sormus de Castro Pinto et al. (2009) with lupin. Alcalase 2.4 L, papain, and pepsin proved to be the most effective enzyme preparation to reduce the abundance of major allergens. Goggin et al. (2008) observed a strong IgE reaction for polypeptides of β‐conglutin >40 kDa and a more weakly for 25–31 kDa; moreover, the major allergen of *L. angustifolius *L. (Lup an 1.0101) is described with the molecular weight of ~55–61 kDa. Treatments with Alcalase 2.4 L, papain, and pepsin hydrolyzed the polypeptides with molecular sizes 27–84 kDa to smaller fragments with molecular sizes below 23 kDa and thus the polypeptides with the most IgE reaction. Polypeptides of β‐conglutin with molecular weights of 12–16 kDa, as present in treatments with Alcalase 2.4 L, papain, and pepsin, showed no IgE reaction according to Goggin et al. (2008). SDS–PAGE results of papain and pepsin cannot be correlated with the observations of DH. The DH following papain and pepsin treatments were relatively low with 2.61% and 3.37%, respectively. For papain, the differences could be potentially due to the interaction between the cysteine residues released during hydrolysis with papain (cysteine endopeptidase) and the OPA reaction components, which react to an unstable, weakly fluorescent product (Chen, Scott, & Trepman, 1979). Similar results were obtained by Meinlschmidt, Schweiggert‐Weisz, et al. (2016); Meinlschmidt, Sussmann, et al. (2016) with soy protein. Enzymatic treatment with Neutrase 0.8 L, Flavourzyme 1000 L, Protease N‐01, Corolase 7089, and Corolase N did not completely hydrolyze the medium molecular weight and high molecular weight subunits. We observed partial hydrolysis of the middle‐molecular weight (27–36 kDa) and high molecular weight polypeptides (41–84 kDa) of both allergens. Neutrase 0.8 L preparation is classified as a neutral, zinc metallo endopeptidase from *Bacillus amyloliquefaciens* that arbitrary hydrolyzes internal peptide bonds, and Protease N‐01 is a serine endoprotease. Based on the SDS–PAGE results, Neutrase 0.8 L and Protease N‐01 do not appear to have sufficient specific substrate in the LPI to completely hydrolyze the polypeptides of the LPI, although Protease N‐01 is also a serine endopeptidase such as Alcalase 2.4 L and Protamex, which achieved better results. The serine endopeptidases have different specific substrate preferences. Flavourzyme 1000 L is a peptidase preparation of two aminopeptidases, two dipeptidyl peptidases, three endopeptidases, and one α‐amylase from the *Aspergillus oryzae* strain ATCC 42149/RIB 40 (Merz et al., 2015). The key enzyme activity is provided by exopeptidases that released amino acids by hydrolysis of the N‐terminal peptide bond. Based on the SDS–PAGE results, hydrolysis of the N‐terminal peptide bond by Flavourzyme 1000 L does not appear to be sufficient to completely hydrolyze the polypeptides of the LPI and to deplete α‐conglutin and β‐conglutin. However, it seems that high molecular weight and medium molecular weight polypeptides could be partially hydrolyzed. Corolase 7089 and Corolase N are both serine and metallo endopeptidases from *Bacillus subtilis* and characterized by the ability to hydrolyze a broad range of substrates. Despite this ability, the results showed that Corolase 7089 and Corolase N were unable to completely cleave the polypeptides. The peptidases may be more capable of hydrolyzing high molecular weight and medium molecular weight polypeptides than low molecular weight polypeptides. Similar SDS–PAGE results were reported by Meinlschmidt, Sussmann, et al. (2016) for soy protein isolate. The enzymes Neutrase 0.8 L, Flavourzyme 1000 L, Protease N‐01, and Corolase 7089 could not completely hydrolyze the high molecular weight subunits of soy protein. With the exception of pepsin conditions, the control samples (without enzyme) showed no change in the molecular weight distributions (data not shown). Bands in the 31–84 kDa range were depleted in the pepsin control presumably due to acid hydrolysis with pH 2.0 (data not shown).

### 3.3 Effects of enzymatic hydrolysis on the technofunctional properties

#### 3.3.1 Protein solubility

The solubility of LPI and its hydrolysates was determined as a function of pH in the range of 4.0 and 9.0 (Table 4).

**Table 4:** Solubility of LPI and LPI hydrolysates at pH range of pH 4.0 and pH 9.0

Protease used for hydrolysis	Protein solubility (%)					
	pH 4.0	pH 5.0	pH 6.0	pH 7.0	pH 8.0	pH 9.0
LPI (not hydrolyzed)	9.7 ± 0.7^a^	7.0 ± 0.0^a^	43.3 ± 0.1^a^	70.7 ± 1.0^a,b^	79.5 ± 1.0^a,c^	80.7 ± 0.7^a,b,d^
Alcalase 2.4 L	75.0 ± 1.9^b^	72.4 ± 1.8^b,f^	80.08 ± 1.5^b^	82.0 ± 2.00^a,b^	85.6 ± 0.2^a,c^	84.8 ± 1.9^a,d^
Papain	45.4 ± 5.9^d,f,h,j^	56.4 ± 0.0^c,f,g,h,i^	66.0 ± 3.0^b,c,h^	70.7 ± 1.00^a,b^	75.9 ± 0.0^a,b,c^	73.9 ± 0.4^a^
Neutrase 0.8 L	44.1 ± 0.6^d,f,h,j^	47.4 ± 1.0^c,d,g,i^	59.2 ± 0.6^c,d,e^	66.3 ± 0.4^b^	67.9 ± 1.0^a,b^	68.6 ± 0.3^b^
Protease N‐01	30.2 ± 1.4^e,f^	34.1 ± 2.1^e^	49.8 ± 0.4^a,d,f^	73.4 ± 1.90^a,b^	79.2 ± 0.4^a,c^	80.3 ± 0.3^a^
Flavourzyme 1000 L	38.0 ± 0.0^f,j^	39.7 ± 1.1^d,e^	46.5 ± 0.3^a,d^	46.9 ± 0.9^c^	49.0 ± 0.1^b^	48.8 ± 0.1^c^
Protamex	64.1 ± 2.0^g^	67.6 ± 2.1^b,c,f^	72.1 ± 2.3^b,c^	79.4 ± 2.60^a,b^	89.9 ± 9.7^c^	83.9 ± 2.7^a^
Corolase 7089	46.0 ± 1.6^h,j^	51.3 ± 3.1^c,d,g,h,i^	62.1 ± 2.0^c,f^	82.7 ± 1.4^a^	87.0 ± 3.4^a,c^	87.3 ± 5.1^d^
Pepsin	53.4 ± 2.7^i^	57.2 ± 4.6^c,g,h^	64.8 ± 9.5^c,g^	69.0 ± 11.4^b^	75.7 ± 8.0^a,c^	82.1 ± 4.2^a^
Corolase N	41.7 ± 2.6^j^	49.0 ± 2.5^c,d,g,i^	60.2 ± 1.8^e,f,g,h^	78.4 ± 4.10^a,b^	80.3 ± 7.7^a,c^	80.2 ± 7.1^a^

Note

The data are expressed as mean ± *SD* (*n* = 4). Values followed by different letters in a column indicate significant differences between groups (*p* < .05).

The maximum solubility of native LPI of 80.7% occurred at pH 9.0 and the minimum of 7% at pH 5.0, which is near to the isoelectric point (pH 4.5) of LPI as described in Bader et al. (2011), Lqari et al. (2005), Piornos et al. (2015), Rodríguez‐Ambriz, Martínez‐Ayala, Millán, and Dávila‐Ortíz (2005). Compared to native LPI, all hydrolysates showed a significant (*p* ˂ .05) increase in solubility under acidic conditions. The Alcalase 2.4 L hydrolysate showed the highest solubility at pH 4.0 (75%) compared to the other ones. Similarly, lupin flour and α‐conglutin treated with Alcalase 2.4 L (Lqari et al., 2005) as well as soy protein isolate treated with various enzymes (Meinlschmidt, Sussmann, et al., 2016) showed an increase in solubility near the isoelectric point. This increase in solubility of the hydrolysates in acidic solutions compared to native LPI may be due to soluble peptides generated by proteolysis (Tsumura et al., 2005). During protein hydrolysis, large insoluble aggregates are cleaved into smaller peptides thus increasing the availability of ionizable groups for interactions with water molecules and enhancing hydration (Qi, Hettiarachchy, & Kalapathy, 1997). Furthermore, with the increase of pH value (pH > 5.0) protein solubility of hydrolysates increase progressively. The Protamex hydrolysate showed maximum solubility (89.9%) at pH 8.0. Surprisingly, Flavourzyme 1000 L hydrolysates showed the lowest increase in protein solubility in the pH range of 4.0 and 6.0 with 38.0% and 46.5%. Above pH 6.0, the protein solubility of the Flavourzyme 1000 L hydrolysates was lower than the protein solubility achieved for the native LPI. Similarly, results for Flavourzyme 1000 L hydrolysis were observed by Purschke et al. (2018) for insect proteins.

#### 3.3.2 Foaming properties

The foaming properties (foam activity, stability, and density) of the hydrolysates are summarized in Table 5.

**Table 5:** Technofunctional properties (foaming properties and emulsifying capacity) of LPI and LPI hydrolysates

Protease used for hydrolysis	Foam activity	Foam stability at 1 hr	Foam density	Emulsifying capacity
	(%)	(%)	(g/L)	(ml/g)
LPI (not hydrolyzed)	980 ± 20^a^	92 ± 0^a^	98 ± 2^a^	620 ± 0^a^
Alcalase 2.4 L	2676 ± 43^b^	96 ± 0^a^	37 ± 1^b,c^	398 ± 5^b,d^
Papain	2912 ± 0^b^	48 ±0^a^	26 ± 0^b,c^	486 ± 31^c,g,h^
Neutrase 0.8 L	1964 ± 136^c^	91 ± 2^a^	39 ± 2^b^	459 ± 22^d,g^
Protease N‐01	2583 ± 25^b^	91 ± 1^a^	38 ± 1^c^	679 ± 16^a,e,i,j,k^
Flavourzyme 1000 L	1206 ± 10^a^	53 ± 40^a^	68 ± 29^a,c^	300 ± 17^f^
Protamex	2521 ± 83^b^	87 ± 6^a^	30 ± 0^b^	500 ± 31^g,h^
Corolase 7089	2056 ± 120^c^	88 ± 2^a^	42 ± 4^b,c^	560 ± 7^a,h,i,k^
Pepsin	3614 ± 29^d^	91 ± 6^a^	25 ± 0^b^	623 ± 20^a,i,j,k^
Corolase N	1919 ± 177^c^	89 ± 2^a^	40 ± 3^b^	653 ± 67^a,j,k^

Note

The data are expressed as mean ± *SD* (*n* = 4). Values followed by different letters in a column indicate significant differences between groups (*p* < .05).

Foams are biphasic colloidal systems with a continuous liquid phase and a dispersing gas phase. Food proteins with the ability to form stable foams can be used to improve the foam properties of food products. The ability to form and stabilize foams depends on environmental parameters such as temperature and pH, as well as the physicochemical properties of proteins such as surface characteristics, degree of denaturation, solubility, segmental flexibility, and the presence or absence of amphiphilic regions, charged residues and polar groups (Lqari et al., 2005; Pozani, Doxastakis, & Kiosseoglou, 2002). To be a good foaming agent, proteins must rapidly adsorb at the air–water interface during bubble formation and must undergo rapid conformational changes and rearrangements (Pozani et al., 2002). In addition, such proteins must be able to form a cohesive viscoelastic film via intermolecular interactions (Pozani et al., 2002). Whey and egg proteins are highly flexible, and they possess hydrophilic groups that align rapidly within the liquid lamellae as well as hydrophobic groups that align with the gas phase. Proteins from plant sources tend to have a rigid structure, so modifications are required to make them suitable for industrial applications, for example, by thermal denaturation, chemical modification, or enzymatic hydrolysis (Raymundo et al., 1998). Lupin protein isolate hydrolysates showed a significant (*p* ˂ .05) increase in foaming activity compared to native LPI (Table 5). Pepsin hydrolysates showed the highest foam activity (3614%), whereas Flavourzyme 1000 L showed the lowest (1206%). Enzymatic hydrolysis also breaks larger polypeptides into smaller peptides, enhancing the foaming activity by allowing rapid diffusion at the air–water interface (Tsumura et al., 2005). Meinlschmidt, Sussmann, et al. (2016) hydrolyzed a soy protein isolate using different enzymes and observed that the foaming activity of the hydrolysates increased with all treatments. We observed that the increase of foaming activity in LPI hydrolysates reflects a change in protein structure that exposed the hydrophilic and polar groups to interactions with the aqueous environment (Qi et al., 1997). The foam stability among the various LPI hydrolysates revealed significant variations with the papain and Flavourzyme 1000 L hydrolysates, showing foam stability values of just 48% and 53%, respectively, whereas all other hydrolysates retained >85% stability after 1 hr. Large peptides with flexible structures have been shown to stabilize foams, but hydrolysis reduces the protein surface coverage required to stabilize the air–water interface which leads to foam collapse in the hydrolyzed protein foams (El‐Adawy, Rahma, El‐Bedawey, & Gafar, 2001). This assumption is supported by our SDS–PAGE profiles, which showed an extensive decrease in the molecular weight of the papain hydrolysates with resulting low foam stability (48%). Interestingly, most of the hydrolysates showed excellent foam stability, which is in contrast to the results reported for a soy protein isolate (Meinlschmidt, Schweiggert‐Weisz, et al., 2016; Meinlschmidt, Sussmann, et al., 2016), rapeseed proteins (Chabanon et al., 2007), and insect protein (Hall et al., 2017; Purschke et al., 2018). The foam density of the LPI hydrolysates was significantly lower than that of native LPI. As expected, the papain and pepsin LPI hydrolysates showed very low foam density due to the extensive hydrolysis by these enzymes. The other samples showed higher values, which may reflect the lower efficiency of hydrolysis.

#### 3.3.3 Emulsifying capacity

The most common emulsions are oil‐in‐water, so emulsions in the food industry are typically made from proteins and lipids combined with aqueous solutions. To determine how hydrolysis would impact the EC of LPI, we compared the ability of LPI and its hydrolysates to form emulsions. As shown in Table 5, the EC of LPI (620 ml/g) was higher than most of the hydrolysates, with the exception of Protease N‐01 (679 ml/g), Corolase N (653 ml/g), and pepsin (623 ml/g). The emulsifying properties of proteins can be improved by exposing hydrophobic groups which enhanced the interactions between proteins and lipids (Qi et al., 1997). El‐Adawy et al. (2001) and Qi et al. (1997) described a direct correlation between the emulsification capacity of proteins and their solubility. More dissolved protein in an emulsion system means more protein in the interface between the oil phase and the continuous phase during emulsification (Qi et al., 1997). As an example, highly soluble hydrolysates, such as those prepared with Corolase N (78.4% solubility at pH 7), also showed a high emulsification capacity (653 ml/g), compared to the much less soluble Flavourzyme 1000 L hydrolysates (46.9% at pH 7) with a low EC of 300 ml/g.

### 3.4 Sensory analysis of the protein hydrolysates

The untreated LPI was evaluated by a trained panel, which determined the intensities of the taste attributes bitter (intensity score on an unscaled 10 cm line = 1.1), salty (0.7), astringent (0.8), metallic (1.8), oatmeal‐like (4.7), earthy, moldy, beetroot‐like (2.2), fatty, cardboard‐like (4.0), grassy (2.1), pea‐like (1.3), and cooked potato‐like (1.7) (Table 6).

**Table 6:** Sensory profile (descriptive analysis) of nonhydrolyzed LPI and LPI hydrolysates

Protease	Bitter	Salty	Astringent	Oatmeal‐like	Fatty, cardboard‐like	Grassy	Cooked potato‐like
LPI	1.1^a^	0.7^a,b^	0.8^a^	4.7^a^	4.0^a^	2.1^a^	1.7^a^
Alcalase 2.4 L	7.2^b^	0.6^a,b^	3.8^a^	1.2^a,b^	1.3^a^	4.2^a^	1.0^a^
Papain	0.9^a^	0.5^a,b^	1.3^a^	1.7^a,b^	1.7^a^	1.8^a^	1.3^a^
Neutrase 0.8 L	1.1^a^	1.3^a,b^	1.2^a^	4.8^a,b^	2.5^a^	2.0^a^	1.9^a^
Protease N‐01	1.3^a^	0.6^a,b^	0.5^a^	3.8^a,b^	2.5^a^	3.5^a^	2.0^a^
Flavourzyme 1000 L	0.7^a^	0.3^a^	0.8^a^	4.0^a^	2.4^a^	1.9^a^	1.8^a^
Protamex	2.4^a,b^	1.1^a,b^	2.4^a^	3.0^a,b^	0.7^a^	3.1^a^	1.3^a^
Corolase 7089	0.5^a^	0.8^a,b^	1.0^a^	2.8^a,b^	1.0^a^	1.0^a^	1.5^a^
Pepsin	2.0^a^	2.2^b^	1.1^a^	0.9^b^	1.2^a^	1.8^a^	1.1^a^
Corolase N	0.8^a^	0.7^a,b^	0.6^a^	3.4^a,b^	0.4^a^	1.0^a^	1.7^a^

Note

The data are expressed as the median values scored on an unstructured 10‐cm line between not noticeable at the left and very strong at the right, based on an evaluation by 10 panelists (*n* = 10). Values followed by different letters in a column indicate significant differences between groups (*p* < .05).

The Alcalase 2.4 L hydrolysates were rated as extremely bitter (7.2) and exhibited an astringent mouthfeel (3.8), which could limit their use in food products. The intensity of bitterness of all other hydrolysates remained similar to the untreated LPI. One of the most significant factors for bitterness is the hydrophobicity of peptides (Maehashi & Huang, 2009). It is postulated that high hydrophobicity tend of the peptides has an intensely bitter taste (Fu et al., 2018). In addition, it appears that the peptide sequence, volume, and spatial structure also exert an effect on the perception of bitter taste (Kim, Yukio, Kim, & Lee, 2008). As the peptide length has been shown to increases, the bitterness is enhanced as the larger peptide chain length can increase the interactions with bitter receptors (Fu et al., 2018; Kim et al., 2008). Moreover, small hydrophobic peptides may lead to bitterness of protein hydrolysates (Matoba & Hata, 1972). The exact MW width for bitter peptides is controversially documented (Fu et al., 2018). Kim and Li‐Chan (2006) reported that hydrophobic bitter peptides of soy protein hydrolysates were smaller than 1 kDa, whereas Cho et al. (2004) describe bitter peptides in the size of 1–4 kDa as more bitter than peptides smaller than 1 kDa. We can state in our study that the extensive hydrolysis with Alcalase 2.4 L corroborated by DH and SDS–PAGE analysis caused peptide chains to break into smaller polypeptides of less than 23 kDa molecular weight and also caused the most intense bitterness.

In addition, the bitterness depends on the DH (Fu et al., 2018). There is a positive correlation between bitterness and DH when DH values are low (Fu et al., 2018; Newman et al., 2014). During hydrolysis, more hydrophobic amino acids are released, resulting in increased bitterness (Spellman, O'Cuinn, & FitzGerald, 2009). However, we could not find a correlation between bitterness and DH. Although the Alcalase 2.4 L hydrolysate exhibited the largest DH and also the highest bitter intensity, the Flavourzyme hydrolysate with a high DH of 6.9% showed a low bitterness of 0.7. The internally located hydrophobic amino acids and peptides, respectively, are more bitter than those located at the N or C terminus of the protein (Matoba & Hata, 1972). This could be the reason that exopeptidases such as Flavourzyme cleaving at the N or C terminus of the proteins and peptides lead to hydrolysates with low bitter intensity despite of the high DH value. In addition, the exopeptidases can selectively cleave peptide bonds at the N or C terminus of bitter peptides, releasing free hydrophobic amino acids and further reducing the bitter taste (Fu et al., 2018).

The pepsin hydrolysate was perceived as more salty (2.2) than LPI and the other hydrolysates due to posthydrolysis neutralization from pH 2 to pH 7 using 3.0 M NaOH. In addition, the intensity of an oatmeal‐like impression decreased to 0.9. In general, treatment with Corolase 7089 and Corolase N improved the sensory profile compared to the native LPI, treatment with Flavourzyme 1000 L, papain, Neutrase 0.8 L, Protease N‐01, and pepsin was acceptable, but treatment with Alcalase 2.4 L induced undesirable changes, including the perception of bitterness, astringency, metallic, earthy or moldy, grassy and pea‐like flavors, as indicated above.

## 4 CONCLUSIONS

The aim of this study was to investigate the effect of enzymatic hydrolysis using different proteases on the integrity of the proteins of LPI by means of DH and molecular weight distribution, their technofunctionality and sensory properties. For an initial allergen assessment, SDS–PAGE and DH revealed that enzymatic hydrolysis can help to reduce the abundance of major allergens. SDS–PAGE results indicated that Alcalase 2.4 L, papain, and pepsin were the most effective proteases, breaking the large polypeptides into low molecular weight peptides. The sensory and technofunctional properties of LPI were improved by most of the proteases, but Alcalase 2.4 L was exceptional and induced primarily undesirable sensory attributes. In order to gain more insight, it will be necessary to compare the allergen structure of LPI and its hydrolysates and to develop more reliable detection methods to quantify the allergens. Further studies should also address the methods to reduce the bitterness of the hydrolysates and therefore improve their potential for use as food ingredients.

## CONFLICT OF INTEREST

The authors declare that they have no conflict of interest.

## ETHICAL APPROVAL

This study does not involve any human or animal testing.

## INFORMED CONSENT

Written informed consent was obtained from all study participants.

## References

[fsn31139-bib-0001] Adler‐Nissen, J. (1986). Enzymic hydrolysis of food proteins. London, UK and New York, NY: Elsevier Applied Science Publishers; Sole distributor in the USA and Canada: Elsevier Science Pub. Co.

[fsn31139-bib-0002] Álvarez‐Álvarez, J. , Guillamón, E. , Crespo, J. F. , Cuadrado, C. , Burbano, C. , Rodríguez, J. , … Muzquiz, M. (2005). Effects of extrusion, boiling, autoclaving, and microwave heating on lupine allergenicity. Journal of Agricultural and Food Chemistry, 53(4), 1294–1298. 10.1021/jf0490145 15713055

[fsn31139-bib-0003] Arnoldi, A. , Boschin, G. , Zanoni, C. , & Lammi, C. (2015). The health benefits of sweet lupin seed flours and isolated proteins. Journal of Functional Foods, 18, 550–563. 10.1016/j.jff.2015.08.012

[fsn31139-bib-0004] Bader, S. , Oviedo, J. P. , Pickardt, C. , & Eisner, P. (2011). Influence of different organic solvents on the functional and sensory properties of lupin (*Lupinus angustifolius* L.) proteins. LWT – Food Science and Technology, 44(6), 1396–1404. 10.1016/j.lwt.2011.01.007

[fsn31139-bib-0005] Ballabio, C. , Peñas, E. , Uberti, F. , Fiocchi, A. , Duranti, M. , Magni, C. , & Restani, P. (2013). Characterization of the sensitization profile to lupin in peanut‐allergic children and assessment of cross‐reactivity risk. Pediatric Allergy and Immunology, 24(3), 270–275. 10.1111/pai.12054 23551124

[fsn31139-bib-0006] Blagrove, R. J. , & Gillespie, J. M. (1975). Isolation, purification and characterization of the seed globulins of *Lupinus angustifolius* . Australian Journal of Plant Physiology, 2(1), 13–27. 10.1071/PP9750013

[fsn31139-bib-0007] Chabanon, G. , Chevalot, I. , Framboisier, X. , Chenu, S. , & Marc, I. (2007). Hydrolysis of rapeseed protein isolates: Kinetics, characterization and functional properties of hydrolysates. Process Biochemistry, 42(10), 1419–1428. 10.1016/j.procbio.2007.07.009

[fsn31139-bib-0008] Chen, R. F. , Scott, C. , & Trepman, E. (1979). Fluorescence properties of o‐phthaldialdehyde derivatives of amino acids. Biochimica Et Biophysica Acta (BBA) – Protein Structure, 576(2), 440–455. 10.1016/0005-2795(79)90419-7 427201

[fsn31139-bib-0009] Chizoba Ekezie, F.‐G. , Cheng, J.‐H. , & Sun, D.‐W. (2018). Effects of nonthermal food processing technologies on food allergens: A review of recent research advances. Trends in Food Science & Technology, 74, 12–25. 10.1016/j.tifs.2018.01.007

[fsn31139-bib-0010] Cho, M. J. , Unklesbay, N. , Hsieh, F.‐H. , & Clarke, A. D. (2004). Hydrophobicity of bitter peptides from soy protein hydrolysates. Journal of Agricultural and Food Chemistry, 52(19), 5895–5901. 10.1021/jf0495035 15366839

[fsn31139-bib-0011] Czubinski, J. , Montowska, M. , Pospiech, E. , & Lampart‐Szczapa, E. (2017). Proteomic analysis of *Lupinus angustifolius* (var. Zeus and Bojar) and *Lupinus luteus* (var. Lord and Parys) seed proteins and their hydrolysates. Journal of the Science of Food and Agriculture, 97(15), 5423–5430. 10.1002/jsfa.8436 28516510

[fsn31139-bib-0012] de Sormus de Castro Pinto, S. E. , Neves, V. A. , & Machado de Medeiros, B. M. (2009). Enzymatic hydrolysis of sweet lupin, chickpea, and lentil 11S globulins decreases their antigenic activity. Journal of Agricultural and Food Chemistry, 57(3), 1070–1075. 10.1021/jf803108c 19170500

[fsn31139-bib-0013] Duranti, M. , Restani, P. , Poniatowska, M. , & Cerletti, P. (1981). The seed globulins of *Lupinus albus* . Phytochemistry, 20(9), 2071–2075. 10.1016/0031-9422(81)80087-8

[fsn31139-bib-0014] El‐Adawy, T. A. , Rahma, E. H. , El‐Bedawey, A. A. , & Gafar, A. F. (2001). Nutritional potential and functional properties of sweet and bitter lupin seed protein isolates. Food Chemistry, 74(4), 455–462. 10.1016/S0308-8146(01)00163-7

[fsn31139-bib-0015] Fu, Y. , Liu, J. , Hansen, E. T. , Bredie, W. L. P. , & Lametsch, R. (2018). Structural characteristics of low bitter and high umami protein hydrolysates prepared from bovine muscle and porcine plasma. Food Chemistry, 257, 163–171. 10.1016/j.foodchem.2018.02.159 29622194

[fsn31139-bib-0016] Goggin, D. E. , Mir, G. , Smith, W. B. , Stuckey, M. , & Smith, P. A. C. (2008). Proteomic analysis of lupin seed proteins to identify conglutin beta as an allergen, Lup an 1. Journal of Agricultural and Food Chemistry, 56(15), 6370–6377. 10.1021/jf800840u 18620408

[fsn31139-bib-0017] Hall, F. G. , Jones, O. G. , O'Haire, M. E. , & Liceaga, A. M. (2017). Functional properties of tropical banded cricket (*Gryllodes sigillatus*) protein hydrolysates. Food Chemistry, 224, 414–422. 10.1016/j.foodchem.2016.11.138 28159288

[fsn31139-bib-0018] Jappe, U. , & Vieths, S. (2010). Lupine, a source of new as well as hidden food allergens. Molecular Nutrition and Food Research, 54(1), 113–126. 10.1002/mnfr.200900365 20013885

[fsn31139-bib-0019] Jimenez‐Lopez, J. C. , Foley, R. C. , Brear, E. , Clarke, V. C. , Lima‐Cabello, E. , Florido, J. F. , … Smith, P. M. C. (2018). Characterization of narrow‐leaf lupin (*Lupinus angustifolius* L.) recombinant major allergen IgE‐binding proteins and the natural β‐conglutin counterparts in sweet lupin seed species. Food Chemistry, 244, 60–70. 10.1016/j.foodchem.2017.10.015 29120805

[fsn31139-bib-0020] Kim, H.‐O. , & Li‐Chan, E. C. Y. (2006). Quantitative structure−activity relationship study of bitter peptides. Journal of Agricultural and Food Chemistry, 54(26), 10102–10111. 10.1021/jf062422j 17177547

[fsn31139-bib-0021] Kim, M.‐R. , Yukio, K. , Kim, K. M. , & Lee, C.‐H. (2008). Tastes and structures of bitter peptide, asparagine‐alanine‐leucine‐proline‐glutamate, and its synthetic analogues. Journal of Agricultural and Food Chemistry, 56(14), 5852–5858. 10.1021/jf7036664 18576657

[fsn31139-bib-0022] Laemmli, U. K. (1970). Cleavage of structural proteins during the assembly of the head of bacteriophage T4. Nature, 227, 680–685. 10.1038/227680a0 5432063

[fsn31139-bib-0023] Lqari, H. , Pedroche, J. , Girón‐Calle, J. , Vioque, J. , & Millán, F. (2005). Production of *Lupinus angustifolius* protein hydrolysates with improved functional properties. Grasas Y Aceites, 56(2), 135–140. 10.3989/gya.2005.v56.i2.121

[fsn31139-bib-0024] Maehashi, K. , & Huang, L. (2009). Bitter peptides and bitter taste receptors. Cellular and Molecular Life Sciences, 66(10), 1661–1671. 10.1007/s00018-009-8755-9 19153652 PMC11115905

[fsn31139-bib-0025] Matoba, T. , & Hata, T. (1972). Relationship between bitterness of peptides and their chemical structures. Agricultural and Biological Chemistry, 36(8), 1423–1431. 10.1080/00021369.1972.10860410

[fsn31139-bib-0026] Meinlschmidt, P. , Schweiggert‐Weisz, U. , Brode, V. , & Eisner, P. (2016). Enzyme assisted degradation of potential soy protein allergens with special emphasis on the technofunctionality and the avoidance of a bitter taste formation. LWT – Food Science and Technology, 68, 707–716. 10.1016/j.lwt.2016.01.023

[fsn31139-bib-0027] Meinlschmidt, P. , Sussmann, D. , Schweiggert‐Weisz, U. , & Eisner, P. (2016). Enzymatic treatment of soy protein isolates: Effects on the potential allergenicity, technofunctionality, and sensory properties. Food Science & Nutrition, 4(1), 11–23. 10.1002/fsn3.253 26788306 PMC4708632

[fsn31139-bib-0028] Melo, T. S. , Ferreira, R. B. , & Teixeira, A. N. (1994). The seed storage proteins from *Lupinus albus* . Phytochemistry, 37(3), 641–648. 10.1016/S0031-9422(00)90331-5

[fsn31139-bib-0029] Merz, M. , Eisele, T. , Berends, P. , Appel, D. , Rabe, S. , Blank, I. , … Fischer, L. (2015). Flavourzyme, an enzyme preparation with industrial relevance: automated nine‐step purification and partial characterization of eight enzymes. Journal of Agricultural and Food Chemistry, 63(23), 5682–5693. 10.1021/acs.jafc.5b01665 25996918

[fsn31139-bib-0030] Monteiro, S. , Freitas, R. , Rajasekhar, B. T. , Teixeira, A. R. , & Ferreira, R. B. (2010). The unique biosynthetic route from lupinus beta‐conglutin gene to blad. PLoS ONE, 5(1), e8542 10.1371/journal.pone.0008542 20066045 PMC2798717

[fsn31139-bib-0031] Morr, C. V. , German, B. , Kinsella, J. E. , Regenstein, J. M. , Buren, J. V. , Kilara, A. , … Mangino, M. E. (1985). A collaborative study to develop a standardized food protein solubility procedure. Journal of Food Science, 50(6), 1715–1718. 10.1111/j.1365-2621.1985.tb10572.x

[fsn31139-bib-0032] Mosse, J. , Huet, J.‐C. , & Baudet, J. (1987). Relationships between nitrogen, amino acids and storage proteins in *Lupinus albus* seeds. Phytochemistry, 26(9), 2453–2458. 10.1016/S0031-9422(00)83852-2

[fsn31139-bib-0033] Newman, J. , Egan, T. , Harbourne, N. , O'Riordan, D. , Jacquier, J. C. , & O'Sullivan, M. (2014). Correlation of sensory bitterness in dairy protein hydrolysates: Comparison of prediction models built using sensory, chromatographic and electronic tongue data. Talanta, 126, 46–53. 10.1016/j.talanta.2014.03.036 24881533

[fsn31139-bib-0034] Nielsen, P. M. , Petersen, D. , & Dambmann, C. (2001). Improved method for determining food protein degree of hydrolysis. Journal of Food Science, 66(5), 642–646. 10.1111/j.1365-2621.2001.tb04614.x

[fsn31139-bib-0035] Phillips, L. G. , Haque, Z. , & Kinsella, J. E. (1987). A method for the measurement of foam formation and stability. Journal of Food Science, 52(4), 1074–1077. 10.1111/j.1365-2621.1987.tb14279.x

[fsn31139-bib-0036] Piornos, J. A. , Burgos‐Díaz, C. , Ogura, T. , Morales, E. , Rubilar, M. , Maureira‐Butler, I. , & Salvo‐Garrido, H. (2015). Functional and physicochemical properties of a protein isolate from AluProt‐CGNA: A novel protein‐rich lupin variety (*Lupinus luteus*). Food Research International, 76, 719–724. 10.1016/j.foodres.2015.07.013 28455057

[fsn31139-bib-0037] Pozani, S. , Doxastakis, G. , & Kiosseoglou, V. (2002). Functionality of lupin seed protein isolate in relation to its interfacial behaviour. Food Hydrocolloids, 16(3), 241–247. 10.1016/S0268-005X(01)00094-7

[fsn31139-bib-0038] Purschke, B. , Meinlschmidt, P. , Horn, C. , Rieder, O. , & Jäger, H. (2018). Improvement of techno‐functional properties of edible insect protein from migratory locust by enzymatic hydrolysis. European Food Research and Technology, 244(6), 999–1013. 10.1007/s00217-017-3017-9

[fsn31139-bib-0039] Qi, M. , Hettiarachchy, N. S. , & Kalapathy, U. (1997). Solubility and emulsifying properties of soy protein isolates modified by pancreatin. Journal of Food Science, 62(6), 1110–1115. 10.1111/j.1365-2621.1997.tb12224.x

[fsn31139-bib-0040] Raymundo, A. , Empis, J. , & Sousa, I. (1998). White lupin protein isolate as a foaming agent. Zeitschrift für Lebensmitteluntersuchung und ‐Forschung A, 207(2), 91–96. 10.1007/s002170050300

[fsn31139-bib-0041] Rodríguez‐Ambriz, S. L. , Martínez‐Ayala, A. L. , Millán, F. , & Dávila‐Ortíz, G. (2005). Composition and functional properties of *Lupinus campestris* protein isolates. Plant Foods for Human Nutrition, 60(3), 99–107. 10.1007/s11130-005-6835-z 16187011

[fsn31139-bib-0042] Sirtori, E. , O'Kane, F. , Brambilla, F. , & Arnoldi, A. (2008). *L. angustifolius* vs *L. albus*: A combined chromatographic and electrophoretic analysis to highlight the differences in protein profile. Paper presented at the Proceedings of the 12th international lupin conference—lupins for health and wealth.

[fsn31139-bib-0043] Spellman, D. , O'Cuinn, G. , & FitzGerald, R. J. (2004). Physicochemical and sensory characteristics of whey protein hydrolysates generated at different total solids levels. Journal of Dairy Research, 72(2), 138–143. 10.1017/S0022029904000688 15909678

[fsn31139-bib-0044] Spellman, D. , O'Cuinn, G. , & FitzGerald, R. J. (2009). Bitterness in Bacillus proteinase hydrolysates of whey proteins. Food Chemistry, 114(2), 440–446. 10.1016/j.foodchem.2008.09.067

[fsn31139-bib-0045] Tsumura, K. , Saito, T. , Kugimiya, W. , & Inouye, K. (2004). Selective proteolysis of the glycinin and beta‐conglycinin fractions in a soy protein isolate by pepsin and papain with controlled pH and temperature. Journal of Food Science, 69(5), C363–C367. 10.1111/j.1365-2621.2004.tb10698.x

[fsn31139-bib-0046] Tsumura, K. , Saito, T. , Tsuge, K. , Ashida, H. , Kugimiya, W. , & Inouye, K. (2005). Functional properties of soy protein hydrolysates obtained by selective proteolysis. LWT – Food Science and Technology, 38(3), 255–261. 10.1016/j.lwt.2004.06.004

[fsn31139-bib-0047] Wang, C. Y. , & Johnson, L. A. (2001). Functional properties of hydrothermally cooked soy protein products. Journal of the American Oil Chemists Society, 78(2), 189–195. 10.1007/s11746-001-0242-y

